# Exercise, Nutrition, and Neuromuscular Electrical Stimulation for Sarcopenic Obesity: A Systematic Review and Meta-Analysis of Management in Middle-Aged and Older Adults

**DOI:** 10.3390/nu17091504

**Published:** 2025-04-29

**Authors:** Shan Xu, Siying Tu, Xiaoyu Hao, Xiangjun Chen, Da Pan, Wang Liao, Ruipeng Wu, Ligang Yang, Hui Xia, Shaokang Wang, Guiju Sun

**Affiliations:** 1 Key Laboratory of Environmental Medicine and Engineering of Ministry of Education, Department of Nutrition and Food Hygiene, School of Public Health, Southeast University, Nanjing 210009, China; 220234094@seu.edu.cn (S.X.); 230248566@seu.edu.cn (S.T.); haoxiaoyu1386@126.com (X.H.); pan_da@seu.edu.cn (D.P.); wangliao@seu.edu.cn (W.L.); yangligang2012@163.com (L.Y.); huixia@seu.edu.cn (H.X.); gjsun@seu.edu.cn (G.S.); 2 Clinical Medical Research Center for Plateau Gastroenterological Disease of Xizang Autonomous Region, School of Medicine, Xizang Minzu University, Xianyang 712082, China; xjchen@xzmu.edu.cn (X.C.); wurp@xzmu.edu.cn (R.W.)

**Keywords:** exercise interventions, nutritional intervention, diet, neuromuscular electrical stimulation, middle-aged and older adults, sarcopenic obesity, meta-analysis

## Abstract

**Background/Objective:** Sarcopenic obesity (SO), a pathological syndrome characterized by the co-existence of diminished muscle mass and excessive adipose accumulation, significantly compromises the quality of life in older adults. The purpose of this study was to systematically evaluate the efficacy of exercise, nutritional interventions, and neuromuscular electrical stimulation (NMES) in preventing and managing SO in middle-aged and older adults. **Methods:** A comprehensive search was conducted across PubMed, Web of Science, Embase, Cochrane Library, and CNKI for randomized controlled trials (RCTs) until January 2025. Meta-analyses were performed using the random-effects model and fixed-effects model based on the degree of heterogeneity and calculating the mean differences (MD) with 95% confidence intervals (CI). Subgroup analyses compared the intervention types. **Results:** Twenty-nine RCTs (1622 participants) were included. Exercise interventions significantly reduced the body fat percentage (MD = −2.79%, 95% CI: −3.94, −1.64, *p* < 0.001, *I*^2^ = 74%), fat mass (MD = −6.77 kg, 95% CI: −11.48, −2.06, *p* = 0.005, *I*^2^ = 98%), waist circumference (MD = −2.05 cm, 95% CI: −3.64, −0.46, *p* = 0.01, *I*^2^ = 0%) and LDL-C (MD: −7.45 mg/dL, 95% CI: −13.82, −1.07, *p* = 0.02, *I*^2^ = 0%), while improving handgrip strength (MD = 2.35 kg, 95% CI: 1.99, 2.70, *p* < 0.001, *I*^2^ = 52%) and gait speed (MD = 0.19 m/s, 95% CI: 0.13, 0.24, *p* < 0.001, *I*^2^ = 89%). Mixed training outperformed resistance-only regimens in reducing the body fat percentage and enhancing functional outcomes. NMES reduced the body fat percentage (MD = −2.01%, 95% CI: −3.54, −0.48, *p* = 0.01, *I*^2^ = 93%) and waist circumference (MD = −1.72 cm, 95% CI: −2.35, −1.09, *p* < 0.001, *I*^2^ = 0%) while increasing the Skeletal Muscle Index (MD = 0.26 kg/m^2^, 95% CI: 0.22, 0.29, *p* < 0.001, *I*^2^ = 38%). Synergy with nutritional supplementation amplified these effects. Nutritional interventions modestly improved total fat-free mass (MD = 0.77 kg, 95% CI: 0.04, 1.50, *p* = 0.04, *I*^2^ = 0%) and handgrip strength (MD = 1.35 kg, 95% CI: 0.71, 2.00, *p* < 0.001, *I*^2^ = 0%) but showed no significant impact on the metabolic markers (TG, TC, glucose, hemoglobin, and HOMA-IR). **Conclusions:** Exercise, particularly multimodal regimens combining aerobic and resistance training, is the cornerstone for improving body composition and physical function in SO. NMES serves as an effective adjunct for accelerating fat loss, while nutritional strategies require integration with exercise or prolonged implementation to yield clinically meaningful outcomes. Future research should prioritize standardized diagnostic criteria and long-term efficacy assessments of multimodal interventions.

## 1. Introduction

Sarcopenic obesity (SO) is a syndrome in which sarcopenia (an age-related loss of muscle mass, muscle strength, and somatic function) coexists with obesity (abnormal accumulation of body fat) and is characterized by the pathology of increased adiposity and decreased skeletal muscle mass and function [1]. The central feature is “high body fat and low muscle mass” rather than being simply overweight. For example, some patients may have a standard body mass index (BMI). Still, their body fat percentage (BF%) is significantly higher, and their muscle mass is far below the healthy threshold, resulting in “hidden obesity” [2].

With increasing age, older adults are prone to SO, a high-risk geriatric syndrome, due to decreased muscle mass and physical activity, leading to decreased metabolism, which, in turn, leads to weight gain and increased abdominal fat [3,4]. At the same time, obesity and especially increased visceral adiposity causes chronic low-grade inflammation and the production of a variety of pro-inflammatory cytokines, such as TNF-α, IL-6, IL-1β, and MCP-1, which, when released into the circulation, promote muscle catabolism, leading to muscle loss [5]. According to a global study, the overall prevalence of SO is approximately 11% in older adults and increases significantly with age, especially in those over 75 years of age, where the prevalence rises to 23% [6]. SO is not the result of a single factor but rather the interplay of multiple elements, including aging [7], hormonal imbalances (decreased growth hormone, testosterone) [8], sedentariness and physical inactivity [9], nutritional imbalances (high-calorie, low-protein diets, vitamin D deficiency) [10], as well as chronic inflammation [11], oxidative stress, and endocrine disorders (e.g., abnormal thyroid function, cortisol) [12].

SO causes a higher risk of metabolic disease and dysfunction, and even death, compared to obesity and sarcopenia alone [13]. Studies have shown that the risk of type 2 diabetes mellitus in SO patients is 1.94 times higher than in healthy people, as fat accumulation exacerbates insulin resistance, and muscle loss further impairs glucose metabolism [14]. A health and aging study demonstrated a 3-fold increased risk of frailty and a 1.5-fold increased risk of disability in SO patients over a 7-year follow-up period [15]. In addition, a cohort study based on older men found that the risk of all-cause mortality in men with SO was approximately 1.22 times that of men with sarcopenia alone [16].

The diagnosis of sarcopenic obesity (SO) follows a two-step protocol comprising screening and diagnostic confirmation, as per the ESPEN/EASO joint criteria established in 2022 [17]. Screening for SO primarily relies on the co-existence of an elevated BMI or waist circumference and sarcopenia-related indicators, such as low skeletal muscle mass and impaired muscle function. Diagnosis involves assessing skeletal muscle functional parameters, including handgrip strength, gait speed, the five-times chair stand test, and the short physical performance battery. If reduced function is observed, body composition should be evaluated through dual-energy X-ray absorptiometry (DXA) or bioelectrical impedance analysis (BIA). The diagnosis is confirmed by the concurrent presence of pathological adiposity and low skeletal muscle mass.

For the prevention and treatment of SO, a combination of interventions is currently used, mainly including exercise intervention, nutritional management, and neuromuscular electrical stimulation (NMES) [18]. Previously, several systematic reviews have investigated interventions for patients with SO to assess the impact of these interventions on their overall health. However, most of the existing studies have focused on single interventions, lacked comprehensive analyses of NMES with multimodal interventions, and have not adequately considered the impact of these interventions on other aspects of health in patients with SO. With this in mind, we conducted a systematic review and meta-analysis aimed at comprehensively assessing the combined effects of exercise, nutrition, and NMES on patients with SO. In addition to the primary outcome measures of SO (e.g., muscle mass, fat mass, grip strength), other physiologic measures (e.g., lipids, blood pressure) were also considered to provide a more informative basis for clinical treatment and health management.

## 2. Materials and Methods

This systematic review was performed according to the Preferred Reporting Items for Systematic Reviews and Meta-Analyses (PRISMA) guidelines and was registered with PROSPERO (CRD420251013148).

### 2.1. Literature Search

Searches were performed in the following databases: PubMed, Web of Science, Embase, Cochrane Library, and CNKI. Relevant studies in English and Chinese published up to January 2025 were searched using the following search terms: (“sarcopenic obese” OR “sarcopenic obesity” OR “sarcopenia obesity” OR “obese sarcopenic” OR “obese sarcopenia” OR “obesity sarcopenia”) AND (“exercise” OR “training” OR “physical”) OR (“food” OR “diet” OR “Nutrition”) OR (“neuromuscular electrical stimulation” OR “electromyostimulation” OR “Electrical Acupuncture” OR “NMES”). Medical subject headings and free text searches were used. Manual reference tracing was performed to identify additional relevant studies.

### 2.2. Inclusion and Exclusion Criteria

Inclusion criteria were based on the PICOS (population, intervention, comparison, outcome, and study design) methodology [19]:

- Population(P): Adults aged ≥ 45 years meeting either (1) a SO diagnosis, (2) being sarcopenic overweight, or (3) obesity with a sarcopenia risk. The exclusion conditions included active neurological/cognitive impairments, severe cardiovascular diseases, acute infection, active autoimmune disease, or malignancy.
- Intervention(I): Exercise protocols (resistance/aerobic training), nutritional optimization (protein supplementation, vitamin D), or neuromuscular electrical stimulation (NMES).
- Comparison(C): Non-pharmacological comparators, usual care, a placebo, or a blank control.
- Outcomes(O): The primary endpoints included body composition (body fat percentage, BMI, waist circumference) and physical performance (gait speed, grip strength). The secondary endpoints included physiologic indicators (blood pressure, lipid profiles).
- Study design(S): Peer-reviewed randomized controlled trials (RCTs) or cluster RCTs.

The exclusion criteria included (1) duplicate publications, (2) unavailable full-text/data, and (3) non-peer-reviewed sources (conference abstracts, dissertations).

### 2.3. Study Selection

Conducted independently by two researchers, the titles and abstracts were screened independently for the inclusion and exclusion criteria. In the event of differences of opinion, a third researcher conducted an independent assessment and resolved any differences.

### 2.4. Data Extraction

The following data were extracted: (1) Study characteristics: authors, year of publication, country/region, and type of study design; (2) participants: sample size and characteristics, such as gender, age, etc.; (3) intervention: description of the intervention, including intervention modality, dosage, frequency, and duration; (4) comparisons: description of the control group; and (5) endpoints and their changes: anthropometric indices, body function indices, and physiological indices.

### 2.5. Risk of Bias Assessment

Two independent researchers conducted bias risk assessments for included studies using the Cochrane Risk of Bias tool (5.1.0) [20]. Discrepancies were resolved through consultation with a third investigator. The domains evaluated encompassed selection bias, performance bias, detection bias, attrition bias, reporting bias, and other potential biases. Studies fulfilling all methodological criteria were classified as low risk. Studies with only 1 noncompliant criterion were classified as a medium risk. Studies that did not meet multiple criteria were considered high-risk and were subsequently excluded from the review.

### 2.6. Statistical Analysis

Statistical analyses were conducted using Review Manager 5.4. Continuous variables were presented as mean differences (MD) with standard deviations (SD); when the SD was unavailable, values were imputed using Cochrane Handbook 5.1.0 conversion algorithms [20]. Heterogeneity was quantified through Cochran’s Q statistic and the *I*^2^ index. Fixed-effects models were applied for low heterogeneity (*I*^2^ < 50%), while random-effects models addressed substantial heterogeneity (*I*^2^ ≥ 50%). The statistical significance threshold was set at α = 0.05 (two-tailed). Publication bias was evaluated through funnel plot asymmetry testing supplemented by Egger’s regression.

### 2.7. Sensitivity Analysis

For the analyses involving at least three studies that demonstrated significant heterogeneity (*I*^2^ ≥ 50%), sensitivity analyses using the leave-one-out method were used to check the robustness of the primary outcome.

## 3. Results

### 3.1. Literature Screening

**Figure 1:**
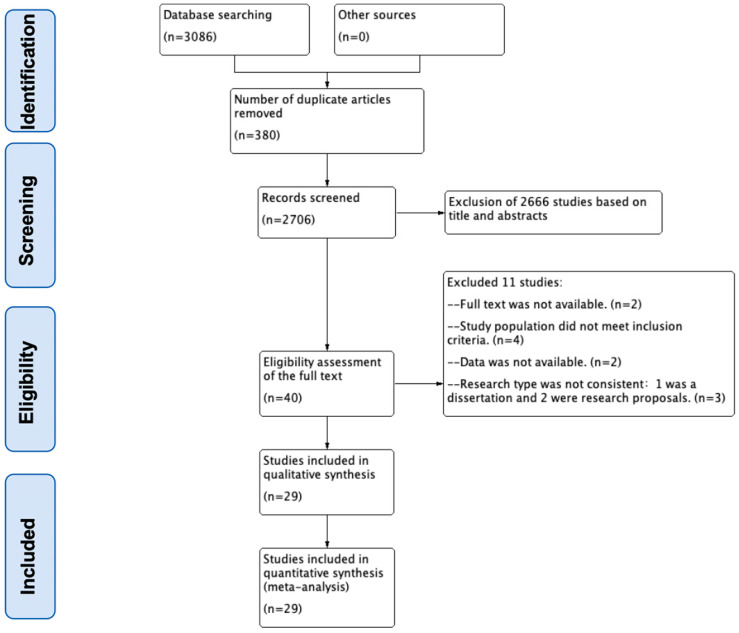
Flow diagram of the study selection procedure.

A total of 3086 articles were identified through the literature search, and 40 articles were included for full-text review after removing the duplicates and reviewing titles and abstracts. After further screening, 29 studies [21,22,23,24,25,26,27,28,29,30,31,32,33,34,35,36,37,38,39,40,41,42,43,44,45,46,47,48,49] were included in the meta-analysis, as shown in Figure 1.

### 3.2. Characteristics of the Included Studies

Table 1 lists the details of the included studies [21,22,23,24,25,26,27,28,29,30,31,32,33,34,35,36,37,38,39,40,41,42,43,44,45,46,47,48,49], including the authors, year of publication, country or region, type of study, participants subgroups, interventions, controls, and outcome indicators. The studies were categorized into exercise, nutrition, and electromyographic stimulation based on the intervention. The included studies were published between 2012 and 2024 and were from China, the United States, Brazil, Taiwan, Iran, Tunisia, South Korea, Portugal, Lebanon, Italy, Mexico, Japan, Spain, and Germany. There were a total of 1622 participants, 1141 females and 481 males.

### 3.3. Description of Diagnostic Criteria for Sarcopenic Obesity

Table 2 summarizes the diagnostic criteria for the SO and body composition assessment methodologies across the included studies. There were significant differences in the definition of SO across studies, mainly in the choice of thresholds for muscle mass (SMI, HS, and GS) and body fat (BMI, BF%, and WC). For the assessment of muscle mass, twelve studies used SMI, six studies used percent skeletal muscle mass, and eight studies used GS and HS as diagnostic criteria for sarcopenia. For the identification of obesity, most studies used a BMI ≥ 30 kg/m^2^, and some used a BMI ≥ 25 or 28 kg/m^2^. Eight studies used the BF%, with thresholds ranging from 25% to 35% for the BF%, and two studies used WC to assess obesity.

### 3.4. Effects of Exercise Interventions on SO

Fifteen studies were included [21,22,23,24,25,26,27,28,29,30,31,32,33,34,35], and the training methods included progressive elastic band resistance training, high-speed circuit training, combined aerobic and resistance training, the total mobility plus program (TMP), and posture, strengthening, and exercise capacity training (PSM). Resistance training programs primarily target the major muscle groups with bench presses, pull-downs, leg stretches, and resistance band exercises. The multimodal intervention integrated aerobic training, progressive resistance loading, and postural stability components. Indicators were divided into anthropometric indicators, physical function indicators, and physiological indicators to analyze the effect of exercise on SO.

#### 3.4.1. Effects of Exercise Interventions on Anthropometric Indicators

**Figure 2:**
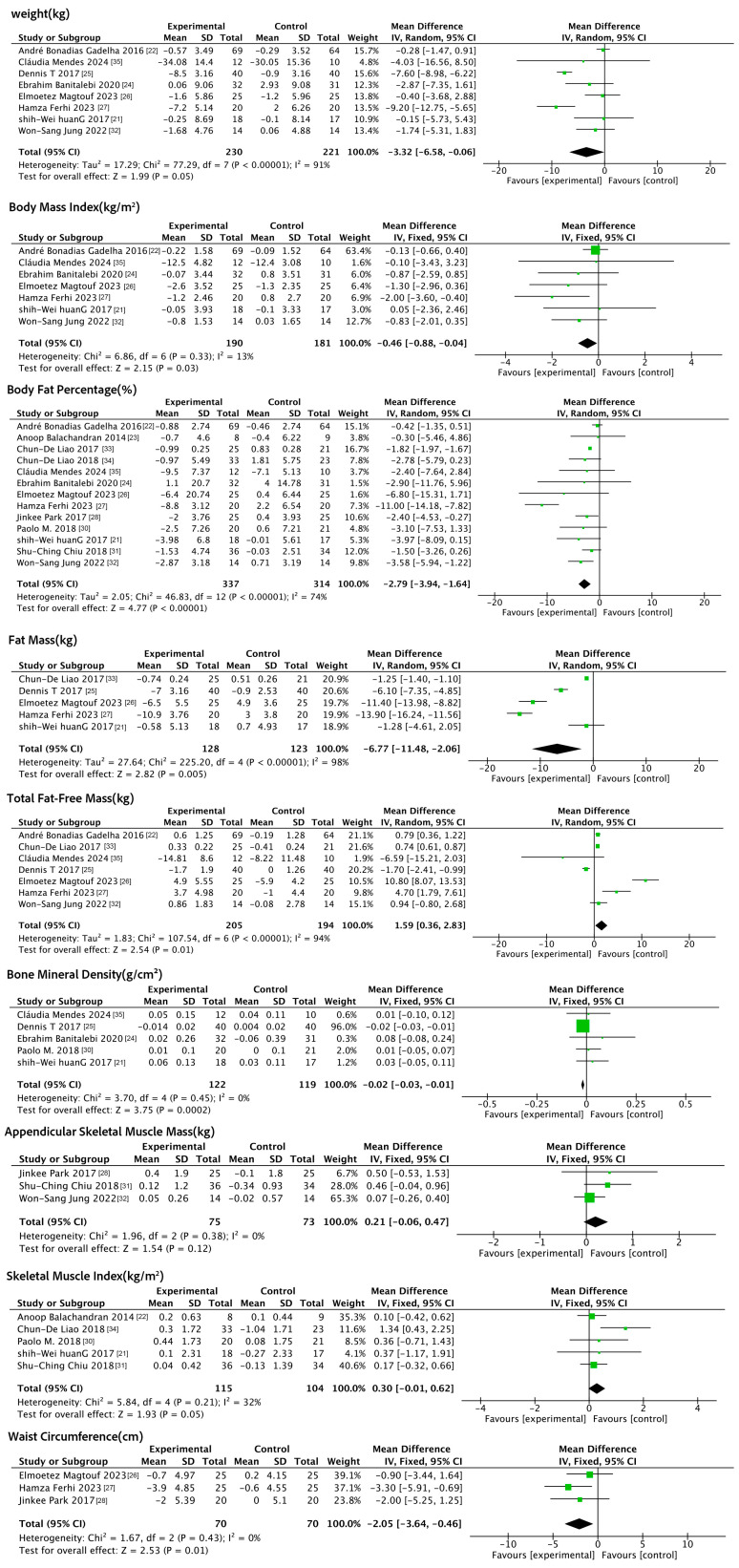
Forest plot of exercise interventions on anthropometric indicators.

Nine anthropometric indicators were included in this analysis, namely weight, BMI, BF%, FM, TFFM, BMD, ASM, SMI, and waist circumference (WC).The forest plot (Figure 2) results showed that, compared to the control group, exercise reduced the BMI (MD: −0.46 kg/m^2^, 95% CI: −0.88, −0.04, *p* = 0.03, *I*^2^ = 13%), BF% (MD: −2.79%, 95% CI: −3.94, −1.64, *p* < 0.001, *I*^2^ = 74%), FM (MD: −6.77 kg, 95% CI: −11.48, −2.06, *p* = 0.005, *I*^2^ = 98%), BMD (MD: −0.02 g/cm^2^, 95% CI: −0.03, − 0.01, *p* < 0.001, *I*^2^ = 0%) and WC (MD: −2.05 cm, 95% CI: −3.64, −0.46, *p* = 0.01, *I*^2^ = 0%) and improved TFFM (MD: 1.59 kg, 95% CI: 0.36, 2.83, *p* = 0.01, *I*^2^ = 94%). In addition, exercise did not significantly improve weight, ASM, or SMI. Improvements in post-exercise weight and the SMI did not reach the preset threshold of significance (*p* = 0.05) but suggest a possible weaker positive trend.

#### 3.4.2. Effects of Exercise Interventions on Indicators of Physical Functioning

**Figure 3:**
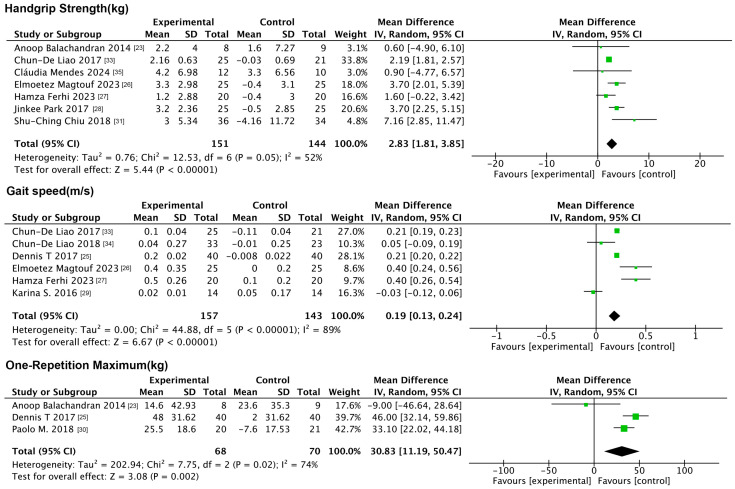
Forest plot of exercise interventions on indicators of physical functioning.

The analysis incorporated three physical function indicators: handgrip strength, gait speed, and total 1 RM. The forest plot (Figure 3) results showed that compared to the control group, exercise significantly increased handgrip strength (MD: 2.35 kg, 95% CI: 1.99, 2.70, *p* < 0.001, *I*^2^ = 52%), gait speed (MD: 0.19 m/s, 95% CI: 0.13, 0.24, *p* < 0.001, *I*^2^ = 89%) and total 1 RM (MD: 30.83 kg, 95% CI: 11.19, 50.47, *p* = 0.002, *I*^2^ = 74%) compared to the controls.

#### 3.4.3. Effects of Exercise Interventions on Physiologic Indicators

**Figure 4:**
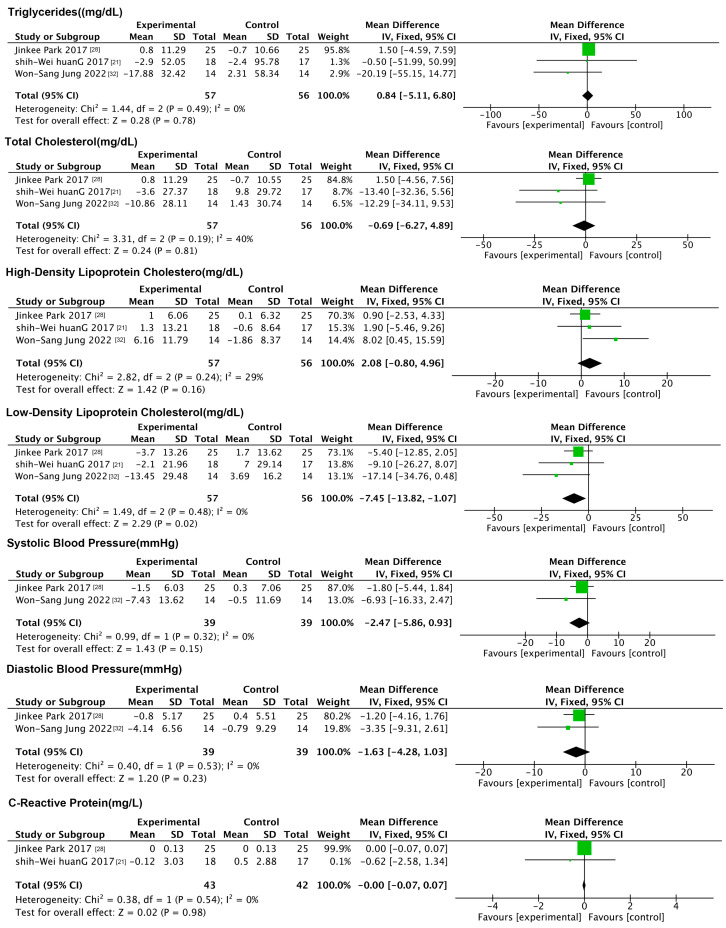
Forest plot of exercise interventions on physiologic indicators.

Seven physiological indicators were included in this analysis, namely the TG, TC, HDL-C, LDL-C, SBP, DBP, and CRP. The forest plot (Figure 4) results showed that exercise reduced only the LDL-C compared to the control group (MD: −7.45 mg/dL, 95% CI: −13.82, −1.07, *p* = 0.02, *I*^2^ = 0%), with no significant changes in the remaining indicators.

### 3.5. Effects of Nutritional Interventions on SO

A total of eight studies were included [36,37,38,39,40,41,42,43], and the interventions included vitamin D supplementation, low-calorie high-protein diets, protein supplements, prebiotics, and probiotics. The indicators are grouped into anthropometric, physical function, and physiological indicators to analyze the impact of nutrition on SO.

#### 3.5.1. Effects of Nutritional Interventions on Anthropometric Indicators

**Figure 5:**
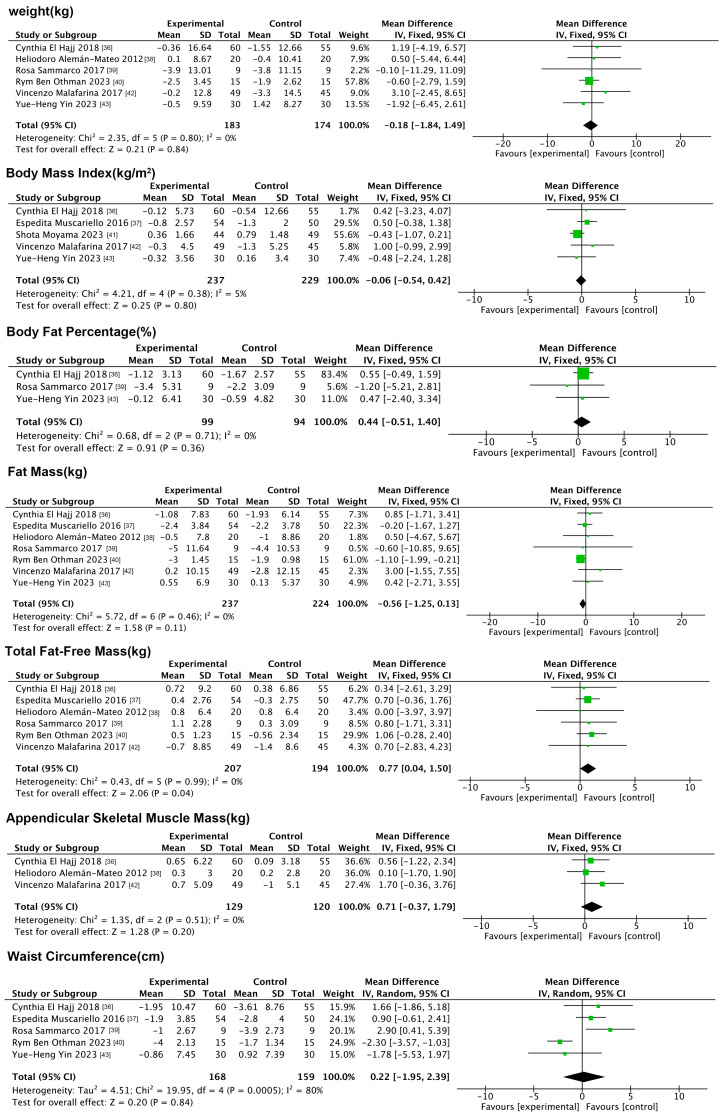
Forest plot of nutritional interventions on anthropometric indicators.

Seven anthropometric indicators were included in this analysis, namely weight, BMI, BF%, FM, TFFM, ASM, and waist circumference. The forest plot (Figure 5) results showed that nutrition only increased TFFM compared to the control group (MD: 0.77 kg, 95% CI: 0.04,1.50, *p* = 0.04, *I*^2^ = 0%). In addition, nutrition did not significantly improve weight, BMI, BF%, FM, ASM, or waist circumference.

#### 3.5.2. Impact of Nutritional Interventions on Indicators of Physical Functioning

**Figure 6:**
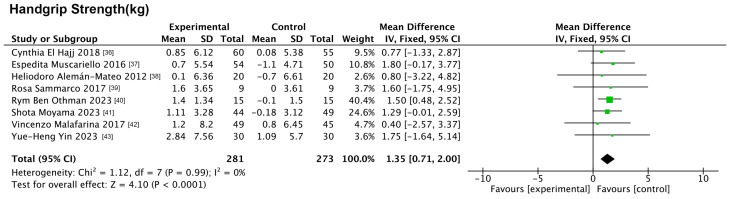
Forest plot of nutritional interventions on indicators of physical functioning.

Only handgrip strength was included in this analysis. The forest plot (Figure 6) results showed that nutrition significantly improved handgrip strength compared to the controls (MD: 1.35 kg, 95% CI: 0.71, 2.00, *p* < 0.001, *I*^2^ = 0%).

#### 3.5.3. Impact of Nutritional Interventions on Physiologic Indicators

**Figure 7:**
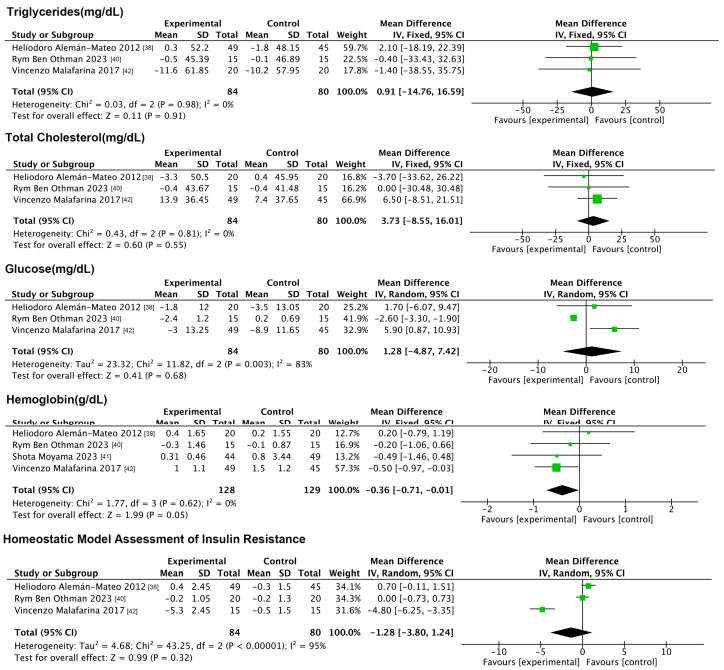
Forest plot of nutritional interventions on physiologic indicators.

Five physiological indices were included in this analysis, namely TG, TC, glucose, hemoglobin, and HOMA-IR. The results of the forest plot (Figure 7) showed that nutrition did not significantly improve any of these indices compared to the control group.

### 3.6. Effects of Neuromuscular Electrical Stimulation on SO

A total of six articles were included [44,45,46,47,48,49], and the interventions included whole-body NMES [44,45] and NMES with nutritional supplementation [46,47,48,49]. In the six studies, the frequency of NMES varied from 1–2 weeks, the duration was 10–20 min, and multiple areas or muscle groups were activated simultaneously with a current frequency of 85 Hz, a pulse width of 350 µs, and the current intensity was adjusted according to the individual. Two of the studies supplemented whey protein powder with vitamin D and essential amino acids, respectively, in conjunction with whole-body NMES. The effects of NMES on SO were analyzed by classifying the indicators into anthropometric, body function, and physiological indicators.

#### 3.6.1. Impact of NMES on Anthropometric Indicators

**Figure 8:**
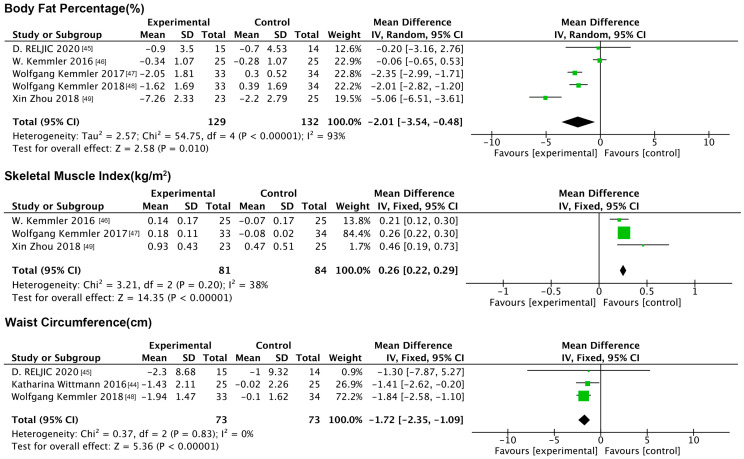
Forest plot of NMES on anthropometric indicators.

Three anthropometric indicators were included in this analysis: BF%, SMI, and waist circumference. The forest plot (Figure 8) results showed that compared to the controls, electromyographic stimulation significantly decreased the BF% (MD: −2.01%, 95% CI: −3.54, −0.48, *p* = 0.01, *I*^2^ = 93%) and waist circumference (MD: −1.72 cm, 95% CI: −2.35, −1.09, *p* < 0.001, *I*^2^ = 0%) and increased the SMI (MD: 0.26 kg/m^2^, 95% CI: 0.22, 0.29, *p* < 0.001, *I*^2^ = 38%).

#### 3.6.2. Impact of NMES on Indicators of Physical Functioning

**Figure 9:**
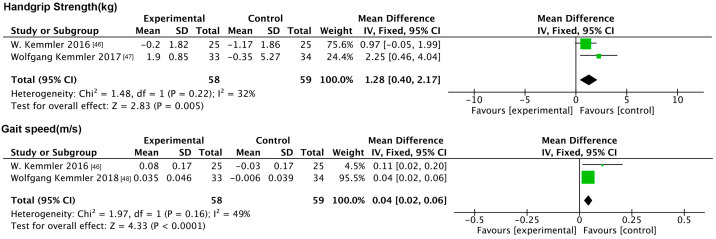
Forest plot of NMES on indicators of physical functioning.

This analysis incorporated handgrip strength and gait speed. The forest plot (Figure 9) results showed that myoelectric stimulation significantly improved handgrip strength (MD: 1.28 kg, 95% CI: 0.40, 2.17, *p* = 0.005, *I*^2^ = 32%) and gait speed (MD: 0.04 m/s, 95% CI: 0.02, 0.06, *p* < 0.001, *I*^2^ = 49%).

#### 3.6.3. Impact of NMES on Physiologic Indicators

**Figure 10:**
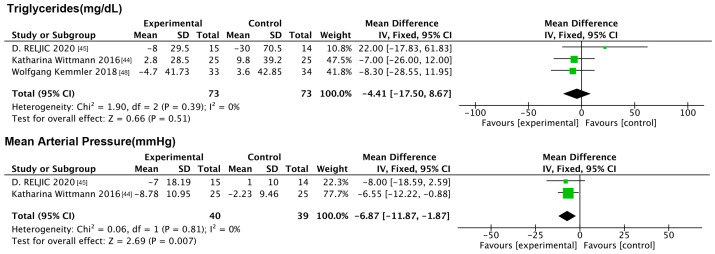
Forest plot of NMES on physiologic indicators.

The analysis incorporated TG and MAB. The forest plot (Figure 10) results showed that myoelectric stimulation improved MAB compared to the controls (MD: −6.87 mmHg, 95% CI: −11.87, −1.87, *p* = 0.007, *I*^2^ = 0%), while it had no improving effect on TG.

### 3.7. Subgroup Analysis

Based on differences in specific interventions, exercise was divided into resistance training alone [21,22,24,29,30,33,34] and mixed training [23,25,26,27,28,31,32,35]; nutritional interventions were divided into high-protein [37,38,39,41,42,43] versus other nutritional interventions (vitamin D, probiotics) [36,40]; and NMES was analyzed in two groups: whole-body NMES [44,45] and supplemental nutritional stimulation [46,47,48,49].

The results showed a prominent effect of the exercise intervention on anthropometric indicators. Mixed training was effective in reducing the BF%, FM, and weight with an MD [95% CI] of −3.85% (95% CI: −6.49, −1.21), −10.39 kg (95% CI: −15.56, −5.22), and −4.79 kg (95% CI: −8.55, −1.02), which were statistically significant (*p* < 0.05). Resistance training alone had a positive effect on elevating the TFFM and SMI, with a mean difference of 0.74 kg (95% CI: 0.62, 0.87) for TFFM and 0.83 kg/m^2^ (95% CI: 0.20, 1.46) for the SMI, both of which were statistically significant (*p* < 0.05). Among the nutritional interventions, the high-protein nutritional intervention (HP) had a limited effect in improving body composition, and the other nutrient interventions (ONS) had mixed results. For NMES, NMES combined with nutritional supplementation (WB-NMES&ns) showed better results in decreasing the BF% and WC and increasing the SMI, all of which were statistically significant (*p* < 0.01).

For the physical function indices, the mixed training was significant in improving HS (MD: 3.19 kg, 95% CI: 2.29, 4.09, *p* < 0.01) and GS (MD: 0.29 m/s, 95% CI: 0.10, 0.48, *p* < 0.01). Resistance training had a limited effect in improving GS but was significant in improving HS and 1 RM (*p* < 0.01). High protein and other nutrient interventions demonstrated similar effects in improving handgrip strength, all of which were statistically significant (*p* < 0.01).

Regarding physiological indicators, all types of interventions had less effect on TG and TC. Nutritional interventions had a more significant effect on GLU, which was increased by the high-protein intervention and decreased by the ONS intervention. The effects of different interventions on Hb and HOMA-IR were limited. Details are given in Table 3.

### 3.8. Biased Risk Assessment

**Figure 11:**
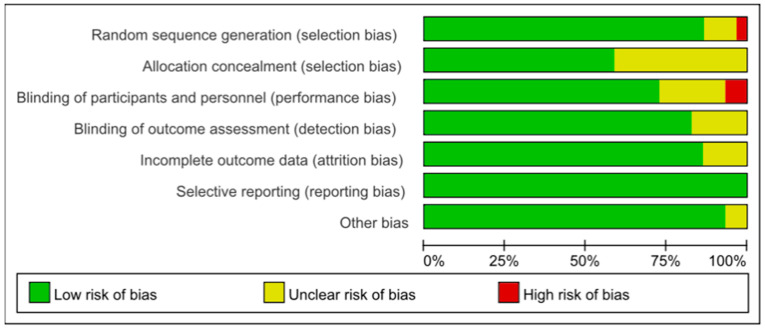
Risk of bias graph.

**Figure 12:**
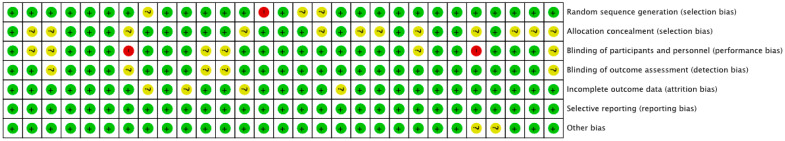
Risk of bias summary [21,22,23,24,25,26,27,28,29,30,31,32,33,34,35,36,37,38,39,40,41,42,43,44,45,46,47,48,49].

Assessed using the Cochrane Risk of Bias Assessment criteria, of the 29 studies, random sequence generation was unknown in three studies, and an inappropriate method of random sequence generation in one study. Allocation concealment was not described in 12 studies; the blinding of participants and personnel was not reported in six studies, and the blinding of participants and personnel was not implemented in two studies. The blinding of the outcome assessment was not reported in five studies; four studies did not report incomplete outcome data; all studies did not report bias, but two studies had other biases. The risk of bias graph (Figure 11) and risk of bias summary (Figure 12) can be seen below.

### 3.9. Publication Bias

**Figure 13:**
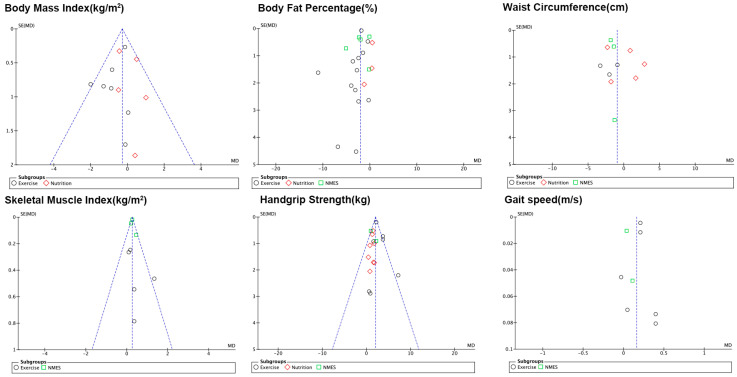
Funnel plot of the BMI, BF%, waist circumference, SMI, handgrip strength, and gait speed.

Publication bias analyses of the main body fat metrics (BMI, BF%, waist circumference) and muscle metrics (SMI, grip strength, step speed) (Figure 13) showed the presence of bias.

### 3.10. Sensitivity Analysis

There was significant heterogeneity in the effects of exercise on the indicators of body weight, BF%, HS, GS, FM, TFFM, and 1 RM. In particular, the effect of exercise on the BF% (*I*^2^ = 74%), HS (*I*^2^ = 52%), GS (*I*^2^ = 89%), and 1 RM (*I*^2^ = 74%) was analyzed by the LOO method and correlated with the Hamza Ferhi, 2023 [27], Shu-Ching Chiu, 2018 [31], Karina S., 2016 [29], and Anoop Balachandran, 2014 [23] studies, and heterogeneity was reduced by excluding these studies, but the conclusions of each study did not change. In addition, regarding the effect of exercise on body weight (*I*^2^ = 91%), FM (*I*^2^ = 98%), and TFFM (*I*^2^ = 94%), the LOO method showed that high heterogeneity remained after excluding studies one by one. After subgroup analysis, the heterogeneity mainly originated from the mixed training subgroup, which may be related to differences in the specific modality, intensity, or frequency of mixed training across studies; these results suggest that differences in the implementation of the mixed training protocols were a central source of heterogeneity for several indicators, whereas the effect of a single study on some of the indicators was significant but did not alter the robustness of the overall findings.

There was significant heterogeneity in the effect of nutrition on waist circumference, fasting GLU, and HOMA-IR. A heterogeneity of 80% for the effect of nutrition on waist circumference, analyzed by the LOO method, was mainly derived from Rym Ben Othman, 2023 [40], while heterogeneity for both fasting GLU (*I*^2^ = 83%) and HOMA-IR (*I*^2^ = 95%) was derived from Vincenzo Malafarina, 2017 [42]. Heterogeneity was reduced by the exclusion of these studies, and there was no substantial change in the results.

The effect of NMES on the BF% was accompanied by significant heterogeneity (*I*^2^ = 93%). The LOO method showed that a high degree of heterogeneity remained after excluding studies one by one. Upon subgroup analysis, the heterogeneity mainly originated from the WB-NMES&ns subgroup and may be related to differences brought about by whole-body NMES accompanied by nutritional supplementation.

## 4. Discussion

In recent years, SO has become an important health challenge in aging societies due to its dual metabolic burden (muscle loss and fat accumulation coexisting). In this study, we comprehensively assessed the preventive and ameliorative effects of exercise, nutrition, and NMES on SO through a systematic review and meta-analysis, revealing the variability of different interventions in improving body composition, function, and physiological indices. Overall, exercise interventions, especially mixed training, showed significant effects in improving anthropometric indices (BMI, BF%) and body function indices (handgrip strength, gait speed). In addition, NMES combined with nutritional supplementation showed better results in reducing body fat and waist circumference. However, nutritional interventions showed relatively limited effects in modulating physiologic indices.

Exercise is a core strategy for improving body composition and function in SO patients. In this study, we found that multimodal training (combining resistance, aerobic, and balance training) was effective in reducing the BMI, BF%, and FM while significantly improving handgrip strength and gait speed. This aligns with Abdollahi et al.’s [50] 12-week randomized trial, where combined high-intensity interval training (HIIT) and resistance protocols elicited superior fat reduction and glycemic control in overweight women through dual mechanisms, such as augmented energy expenditure and activated muscle protein synthesis. Chen et al. [51] also found that mixed training demonstrated a more significant effect in enhancing physical function in SO patients compared to aerobic training. The subgroup analysis showed that although resistance training alone was effective in increasing muscle mass, it was less effective than mixed training in reducing the BF% and visceral fat (waist circumference), suggesting that it mainly promoted muscle fiber thickening by increasing muscle loading and was more focused on muscle mass enhancement than whole-body metabolic regulation [52]. In addition, the weaker effect of resistance training on GS than that of mixed training may be related to the insufficient stimulation of lower limb explosive power and multi-joint coordination, and Wang [53] showed that resistance training would result in the weakening of muscle strength and attenuation of movement speed at specific joint angles, which was not conducive to the development of explosive power; at the same time, its uni-joint dominant training mode also limited the improvement of multi-joint coordination, which were the strengths of mixed training. It is worth pointing out that mixed training was associated with a slight decrease in bone mineral density (BMD), a result that differs from some of the findings in the literature that “high-intensity resistance combined with impact exercise improves BMD in postmenopausal women” [54], which may be due to the lack of mechanical stimulation of the skeleton from aerobic exercise; resistance training stimulates the skeleton, but the intensity and frequency of training may not be sufficient to maintain BMD, and weight loss may also have an effect on BMD.

Mechanistically, aging is closely related to mitochondrial dysfunction and oxidative stress [55]. With age, the mitochondrial DNA mutation rate increases significantly while the activity of electron transport chain complex IV decreases, leading to a decrease in the efficiency of ATP synthesis and a burst accumulation of ROS [56]. Such changes not only weaken the cell’s energy metabolism capacity but also further exacerbate oxidative stress and drive the aging process. Studies have shown that aerobic exercise improves mitochondrial morphology and function, enhances antioxidant capacity, promotes fat oxidation, and improves energy metabolism efficiency through the activation of regulatory factors such as PGC-1α [57,58]. Resistance training, on the other hand, enhances skeletal muscle mitochondrial remodeling and muscle protein synthesis, mainly through the mTOR pathway. The mTOR pathway plays a key role in the regulation of muscle growth and repair [59], and resistance training promotes muscle fiber hypertrophy and strength increase by stimulating this pathway [60]. The synergy between the two can balance catabolism and anabolism and realize the dynamic balance between fat loss and muscle growth.

The effect of nutritional intervention on SO patients was selective. Although nutritional intervention increased TFFM and handgrip strength, there was no significant improvement in weight, as well as the BMI, BF%, and physiologic indices. This suggests that nutritional intervention is effective in enhancing body composition and muscle strength but is not effective in lowering body fat. This result is consistent with the finding of Christos et al. [61] that nutritional supplementation alone is difficult to break through the metabolic adaptation threshold and needs to be combined with exercise (e.g., resistance training or aerobic exercise) to activate muscle protein synthesis and fat oxidation. Of note, some studies have shown that high-protein diets may transiently elevate blood glucose, possibly related to enhanced protein gluconeogenesis [62], suggesting the need for individualized adjustment of protein intake and source (e.g., plant or whey protein). Other nutritional strategies (vitamin D, prebiotics) had limited improvement in metabolic markers, but prebiotic interventions lowered glycemia, possibly related to gut flora modulation [63]. Of course, it is also possible that this variability stems from the limited sample size and the short duration of the intervention (only 1–6 months). Overall, nutritional interventions need to be implemented over a long period of time (>6 months) and combined with exercise to achieve synergistic optimization of the body’s composition and metabolism.

Neuromuscular electrical stimulation (NMES) mimics exercise signals through high-frequency currents, bypassing central neural control to directly activate type II muscle fibers and simulating resistance exercise effects [64]. In this study, we showed that NMES could simultaneously reduce the BF% and WC while elevating the SMI and improving muscle function indexes such as HS and GS, realizing the dual benefits of fat loss and muscle gain, which is consistent with the results of several studies [65,66]. The mechanism may involve (i) electrical stimulation induces muscles to release myokines such as IL-6, which specifically promotes visceral fat lipolysis [67] but has no significant effect on circulating lipids (TG, TC), suggesting the limitation of its regulation of systemic lipid metabolism [68]; and (ii) enhances the efficiency of muscle recruitment through the activation of α-motor neurons in the spinal cord, which improves the functional indexes in the short term [69]. Notably, subgroup analysis showed that NMES combined with nutritional supplementation was more effective than stimulation alone, suggesting that nutritional synergism may enhance the intervention effect [70], and the study by Avelino et al. [71] also demonstrated that the intervention mode combining NMES and high-protein nutritional supplementation could significantly reduce lower limb muscle loss and increase muscle volume and cross-sectional area. It is important to note that although NMES can locally activate muscles, it cannot completely replace the regulatory effects of active exercise on cardiorespiratory function and systemic metabolism [72]. The mechanism of this difference was verified in a study by Mizuki et al. [73], where moderate-intensity active exercise significantly shortened the reaction time (RT) by enhancing sympathetic activity, whereas the NMES intervention did not show similar cognitive improvement effects. This suggests that active exercise systematically activates the autonomic regulatory network, whereas NMES triggers local muscle contraction only through peripheral electrical stimulation and lacks the overall metabolic modulatory effect of central–peripheral linkage.

Although this study integrated existing evidence, limitations remain. First, the included studies were heterogeneous in terms of intervention modality, duration, and assessment metrics, which may have affected the comparison and interpretation of the results. Second, some of the studies were at risk of bias (e.g., unknown methods of randomization, missing blinding), which weakened the robustness of the conclusions. In addition, the lack of long-term follow-up data made it difficult to assess the persistence of the intervention effect. Future studies need to focus on the following directions: (1) developing uniform diagnostic criteria for SO to reduce inter-study heterogeneity; (2) conducting RCTs with large samples and long-term follow-up to clarify the dose–effect relationship of different interventions; and (3) exploring personalized intervention strategies, such as customized exercise–nutritional combination protocols based on baseline body composition and metabolic status.

## 5. Conclusions

Taken together, exercise (especially mixed training) is the core strategy for improving body composition and function in SO patients. NMES can be used as an adjunct to accelerate body fat reduction, and nutritional interventions need to be implemented in conjunction with exercise or over a long period of time to be effective. A multimodal intervention program is recommended to balance muscle mass enhancement and metabolic regulation. Multimodal interventions should be incorporated into community health management guidelines, prioritizing coverage of older SO at-risk populations.

## Figures and Tables

**Table 1:** Characteristics of the included literature.

Author (Year)	Country/Area	Type of Experiment	Participants	Subgroups and Numbers	Intervention	Comparison	Duration	Outcomes
Exercise								
Shih-Wei Huang et al. (2017) [21]	Taiwan	Randomized single-blind (assessor-blind) controlled trial	35 SO patients (aged ≥ 60; all females)	IG: 18; CG: 17	Progressive elastic band training 3 times a week for 55 min (with warm-up, training, and cool-down).	Received only one health education with no follow-up exercise intervention	12 weeks	Weight, BMI, SMI, BF%, TG, HDL, LDL, CRP
André Bonadias Gadelha et al. (2016) [22]	Brazil	Randomized controlled trial	133 SO patients (aged 60–80; all females)	IG: 69; CG: 64	Resistance training program, 3 times a week	Maintaining daily habits, focusing on diet and exercise	24 weeks	Weight, BMI, BF%, TFFM, AFFM
Anoop Balachandran et al. (2014) [23]	America	Randomized controlled single-blind trial	17 SO patients (aged 60–90; M/F: 16/1)	IG: 8; CG: 9	High-speed circuit training, 2 times per week	15 weeks of traditional strength training 2×/week	15 weeks	SPPB, leg press power and 1 RM, chest press power and 1 RM, GS, BF%, SMI
Ebrahim Banitalebi et al. (2020) [24]	Iran	Randomized controlled trial	63 SO patients (aged 65–80, all females)	IG: 32; CG: 31	Resistance training with elastic bands	Maintaining daily eating and activity habits	12 weeks	weight, BMI, BF%, BMC, BMD
Dennis T et al. (2017) [25]	America	Randomized controlled trial	80 obese sedentary older adults (aged ≥ 65; M/F: 57/103)	IG: 40; CG: 40	weight management program + combined training	No weight management or exercise	26 weeks	Weight, LM, FM, TF, BMD at total hip, Total 1 RM, HS, SF-36
Elmoetez Magtouf (2023) [26]	Tunis	Single-blind, prospective, controlled, randomized multicenter trials	50 SO patients (aged > 65, not mentioned)	IG: 25; CG: 25	Participate in the 4-month TMP program three times a week, including motor skill exercises, strengthening exercises, and posture exercises.	Maintaining daily eating and activity habits	4 months	Weight, BMI, LBM
Hamza Ferhi et al. (2023) [27]	Tunis	Single-blind, multicenter, randomized controlled trials	40 SO patients (aged > 65, not mentioned)	IG: 20; CG: 20	Posture, strengthening, and motricity (PSM) training program, twice a week.	Maintenance of daily activities without intervention	24 weeks	BMI, LBM, Maximal HS
Jinkee Park et al. (2017) [28]	South Korea	Randomized single-blind controlled community trials	50 SO patients (aged ≥ 65; all females)	IG: 25; CG: 25	Combined aerobic and resistance training	Maintain daily activities and receive health education	24 weeks	BMI, BF%, ASM, WC, TC, TG, HDL-C, LDL-C, CRP, SBP, DBP
Karina S et al. (2016) [29]	Brazil	Prospective Registry, Two-Arm, Randomized Controlled Trial	28 obese and at risk of sarcopenia older adults (aged 65–80; all females)	IG: 14; CG: 14	Lower extremity resistance exercise program	Weekly telephone monitoring of health status	10 weeks	Knee extension strength, power, HS, SF-36
Paolo M et al. (2018) [30]	Brazil	Randomized controlled trial	41 SO patients (aged ≥ 60, all females)	EG: 20; CG: 21	Three sets of resistance training 3 times per week	No physical exercise activities	12 weeks	Total strength, BF%, BMD
Shu-Ching Chiu et al. (2018) [31]	Taiwan	Randomized controlled trial	70 obese and at risk of sarcopenia older adults (aged ≥ 60; M/F: 35/35)	IG: 36; CG: 36	Resistance training in a sandbag chair 2 times a week	Receive routine care with no additional training	12 weeks	ASM, ASMI, BF%, HS
Won-Sang Jung et al. (2022) [32]	South Korea	Randomized controlled trial	28 SO patients (aged ≥ 65, all females)	IG: 14; CG: 14	Circuit training, 3 times per week	Maintenance of daily activities without sports training	12 weeks	BMI, FM, ASM, SBP, HDL-C, HOMA-IR, CRP, IL-6, IGF-1
Chun-De Liao et al. (2017) [33]	Taiwan	Prospective randomized controlled trials	46 SO patients (aged 60–80, all females)	IG: 25; CG: 21	Elastic resistance training	No exercise intervention	12 weeks	TFFM, TFM, BF%, HS
Chun-De Liao et al. (2018) [34]	Taiwan	Prospective randomized controlled trials	56 SO patients (aged 60–80, all females)	IG: 33; CG: 23	Elastic band resistance training	No exercise intervention	12 weeks	BF%, AMM, SMI, AMI, SF-36,
Cláudia Mendes et al. (2024) [35]	Portugal	Randomized controlled trial	22 SO patients (aged 48–60, M/F: 18/4)	IG: 12; CG: 10	Cardio and strength training 3 times per week	Usual standard of care	16 weeks	Weight, BMI, BF, HS, LM, BMC, BMD
Nutrition								
Cynthia El Hajj et al. (2018) [36]	Lebanon	Randomized, controlled, double-blind trials	115 Pre-oligomyosis patients (mean age 73.31 ± 2.05 years; M/F: 62/66)	IG: 60; CG: 55	10,000 IU of vitamin D supplements 3 times a week	Placebo 3 times per week	6 months	ASMM, BMI, FM, WC, LBM, HS
Espedita Muscariello et al. (2016) [37]	Italy	Randomized controlled trial	104 SO patients (aged ≥ 65; all females)	IG: 54; CG: 50	Dietary intake of 1.2 g of protein per kg of ideal body weight per day	Dietary intake of 0.8 g of protein per kg of ideal body weight per day	3 months	BMI, WC, FM, HS
Heliodoro Alemán-Mateo et al. (2012) [38]	Mexico	Randomized controlled trial	40 SO patients (aged ≥ 60; M/F: 17/23)	IG: 20; CG: 20	Additional 210 g of ricotta cheese per day	Maintained a daily diet with no additional interventions	3 months	Strength, IGF-1, insulin, HOMA-IR, BF%, LBM, weight, glucose, Hb, TG, TC,
Rosa Sammarco et al. (2017) [39]	Italy	Randomized controlled trial	18 SO patients (aged 45–74; all females)	IG: 9; CG: 9	Low-calorie, high-protein diet (1.2–1.4 g/kg /day)	Low-calorie diet + placebo	4 months	Weight, FFM, FAT (% and kg), WC, HS, SPPB, SF-36
Rym Ben Othmale et al. (2023) [40]	Tunis	Randomized controlled trial	30 SO patients (aged ≥ 45; M/F: 3/42)	IG: 15; CG: 15	Low-carb, low-energy diet + 30 g carob/day	Low-carb, low-energy diet only	1 month	BMI, WC, FM, SBP, Hb, HOMA-I, HDL, LDL, TG
Shota Moyama et al. (2023) [41]	Japan	Multicenter, open-label, two-arm, randomized, controlled clinical trial	93 oligomyosis patients (aged ≥ 75; M/F: 51/42)	IG: 44 CG: 49	Dietary intake of 1.5 g of protein per kg of ideal body weight per day	Dietary intake of 1.0 g of protein per kg of ideal body weight per day	6 months	BM, SMI, HS, HB, CRP
Vincenzo Malafarina et al. (2017) [42]	Spanish	Multicenter, randomized, open-label trial	92 oligomyosis patients (aged ≥ 65; M/F: 22/70)	IG: 49; CG: 43	Standard diet + HMB-enriched oral nutritional supplement	Standard diet only	6 weeks	BMI, HS, ASMM, FM, CRP, IL-6, TNF-α, HOMA-IR,
Yue-Heng Yin et al. (2023) [43]	China	Prospective, two-arm, assessor-blinded, parallel-group, pilot randomized controlled trial	60 SO patients (aged ≥ 60; M/F: 18/42)	IG: 30; CG: 30	Eating Behavior Change Intervention Programe	Receive regular health-related lectures	15 weeks	BMI, SMI, HS, WC, 6-m GS, SPPB, SF-36,
Electromyostimulation								
Katharina Wittmalen et al. (2016) [44]	Germany	Single-center, controlled, semi-blind trial, parallel-group design with stratification and randomization	50 SO patients (aged ≥ 70; all females)	IG: 25; CG: 25	WB-NMES,1 time per week for 20 min	Maintained a regular lifestyle.	6 months	WC, HDL-C
D. RELJIC et al. (2020) [45]	Germany	Randomized controlled trial	29 central obesity patients (aged ≥ 50; all females)	IG: 15; CG: 14	2×/week WB-NMES, 20 min/session	No intervention	12 weeks	BM, BF%, TFFM, maximum muscle strength, SBP, DBP, TG
W. Kemmler et al. (2016) [46]	Germany	Single-center, controlled, semi-blind trial, parallel-group design with stratification and randomization	50 SO patients (aged ≥ 70; all females)	IG: 25; CG: 25	WB-NMES (1 time per week for 20 min) + 40 g nutritional supplement daily	Maintained a regular lifestyle.	26 weeks	SMI, HS, BF%
Wolfgang Kemmler et al. (2017) [47]	Germany	Randomized controlled trial, parallel-group design	67 SO patients (aged ≥ 70; all males)	IG: 33; CG: 34	WB-NMES + daily whey protein powder supplements	No intervention	16 weeks	SMI, HS, BF%
Wolfgang Kemmler et al. (2018) [48]	Germany	Randomized controlled trial, parallel-group design	67 SO patients (aged ≥ 70; all males)	IG: 33; CG: 34	WB-NMES + daily whey protein powder supplements	No intervention	16 weeks	BFFM, WC, TC/HDL-C
Xin Zhou et al. (2018) [49]	China	Randomized controlled trial	48 SO patients (aged 60–80; all males)	IG: 24; CG: 24	NMES (20 min every 3 days) + oral essential amino acids (20 g/day) twice daily.	Essential amino acids administered orally twice daily (20 g/day)	28 weeks	BF%, SMI

SO: sarcopenic obesity; IG: intervention group; CG: control group; WB-NMES: whole-body neuromuscular electrical stimulation; 1 RM: one-repetition maximum; AFFM: appendicular fat-free mass; ASM: appendicular skeletal muscle mass; ASMI: Appendicular Skeletal Muscle Mass Index; BMC: bone mineral content; BMD: bone mineral density; BF%: body fat percentage; BMI: Body Mass Index; CRP: C-reactive protein; DBP: diastolic blood pressure; FM: fat mass; HS: handgrip strength; HB: hemoglobin; HDL: high-density lipoprotein; HOMA-IR: homeostatic model assessment of insulin resistance; IGF-1: Insulin-like Growth Factor 1; IL-6: Interleukin-6; LBM: lean body mass; LDL: Low-Density Lipoprotein; SBP: systolic blood pressure; SF-36: 36-Item Short Form Health Survey; SMI: Skeletal Muscle Index; SPPB: short physical performance battery; TC: total cholesterol; TG: Triglycerides; TFFM: total fat-free mass; WC: waist circumference.

**Table 2:** Diagnostic criteria and assessment methods for SO in the included study.

Author (Year)	Diagnostic Criteria for Sarcopenic Obesity		Body Composition Assessment Method
	Assessment of Muscle	Assessment of Body Fat	
Shih-Wei Huang et al. (2017) [21]	ASM/weight < 27.6%	BMI > 30 kg/m^2^	DX, BIA
André Bonadias Gadelha et al. (2016) [22]	Minimal physical activity, risk of sarcopenia	BMI ≥ 30 kg/m^2^	DXA
Anoop Balachandran et al. (2014) [23]	SMI (male < 10.76 kg/m^2^, female < 6.76 kg/m^2^)	BMI ≥ 30 kg/m^2^	BIA
Ebrahim Banitalebi et al. (2020) [24]	10 m walking test: GS ≤ 1 (m/s)	BMI > 30 kg/m^2^	DXA
Dennis T et al. (2017) [25]	Mild to moderate weakness; Physical Performance Test (PPT) scores between 18 and 31	BMI ≥ 30 kg/m^2^	DXA
Elmoetez Magtouf (2023) [26]	HS < 17 N and GS < 1.0 m/s	BMI > 30 kg/m^2^	BIA
Hamza Ferhi et al. (2023) [27]	HS < 17 N and GS < 1.0 m/s	BMI > 30 kg/m^2^	BI, DXA
Jinkee Park et al. (2017) [28]	ASM/weight < 25.1%	BMI ≥ 25 kg/m^2^	BIA
Karina S et al. (2016) [29]	HS ≤ 21 kg	BMI > 30 kg/m^2^	BIA
Paolo M et al. (2018) [30]	Minimal physical activity, risk of sarcopenia	BMI > 30 kg/m^2^	DXA
Shu-Ching Chiu et al. (2018) [31]	ASM/weight (male < 37.15%, female < 32.26%)	BF% (male ≥ 29%, female ≥ 40%) or BMI (25.4–26.1) kg/m^2^	BIA
Won-Sang Jung et al. (2022) [32]	SMI ≤ 5.4 kg/m^2^	BMI ≥ 32 kg/m^2^	DXA
Chun-De Liao et al. (2017) [33]	ASM/weight < 27.6%	BF% > 30%	DXA
Chun-De Liao et al. (2018) [34]	ASM/weight < 27.6%	BF% > 30%	DXA
Cláudia Mendes et al. (2024) [35]	Low muscle strength: HS (male < 27 kg, female < 16 kg; Low muscle mass: ASM/weight (male < 28.27%, female < 23.47%)	Male: BMI ≥ 26 kg/m^2^ Female: BMI ≥ 30 kg/m^2^	DXA
Cynthia El Hajj et al. (2018) [36]	SMI (men < 7.26 kg/m^2^, female < 5.45 kg/m^2^)	BMI ≥ 30 kg/m^2^	BIA
Espedita Muscariello et al. (2016) [37]	SMI < 7.3 kg/m^2^	BMI ≥ 30 kg/m^2^	BIA
Heliodoro Alemán-Mateo et al. (2012) [38]	SMI < 7.89 kg/m^2^	BMI > 30 kg/m^2^	DXA
Rosa Sammarco et al. (2017) [39]	FFM < 90% of ideal FFM	BF% > 34.8%	BIA
Rym Ben Othmale et al. (2023) [40]	Weak muscle strength and risk of sarcopenia	BMI ≥ 30 kg/m^2^	BIA
Shota Moyama et al. (2023) [41]	SMI (male < 7.0 kg/m^2^, female < 5.7 kg/m^2^); HS (male < 28.0 kg, female < 18.0 kg)	BMI ≥ 30 kg/m^2^	BIA
Vincenzo Malafarina et al. (2017) [42]	SMI (female ≤ 5.67 kg/m^2^, male ≤ 7.25 kg/m^2^); GS < 0.8 m/s; HS (female < 20 kg, male < 30 kg)	At risk of obesity	BIA
Yue-Heng Yin et al. (2023) [43]	HS (male ≤ 28 kg, female < 18 kg); 5 chair sit-up tests in ≥12 s	BMI ≥ 28 kg/m^2^; WC: male ≥ 85 cm, female ≥ 80 cm	BIA
Katharina Wittmalen et al. (2016) [44]	SMI < 5.75 kg/m^2^	BF% > 35%	DX, BIA
D. RELJIC et al. (2020) [45]	Habitually sedentary, at risk for sarcopenia	WC > 80 cm	BIA
W. Kemmler et al. (2016) [46]	SMI < 5.75 kg/m^2^	BF% > 35%	DXA, BIA
Wolfgang Kemmler et al. (2017) [47]	SMI < 7.89 kg/m^2^	BF% > 27%	BIA
Wolfgang Kemmler et al. (2018) [48]	SMI < 7.89 kg/m^2^	BF% > 27%	BIA
Xin Zhou et al. (2018) [49]	SMI < 7.0 kg/m^2^	BF% ≥ 25%	BIA

ASM: appendicular skeletal muscle mass; BF%: body fat percentage; BMI: Body Mass Index; FFM: fat-free mass; GS: gait speed; HS: handgrip strength; SMI: Skeletal Muscle Index; WC: waist circumference; BIA: bioelectrical impedance analysis; DXA: dual-energy x-ray absorptiometry.

**Table 3:** Subgroup analysis according to different intervention modalities.

Indicators	Intervention	Subgroup	RCTs (Participants)	MD [95% CI]	*p*	*I*^2^ (%)	Inter-Group *p*-Values	Inter-Group *I*^2^ (%)
Anthropometric indicators								
BF%	exercise	RT	6 (374)	−1.61 [−2.59, −0.64]	<0.01	52	0.12	58.8
		MT	7 (277)	−3.85 [−6.49, −1.21]	<0.01	80		
	nutrition	HP	2 (78)	−0.09 [−2.43, 2.24]	0.94	0	0.62	0
		ONS	1 (115)	0.55 [−0.49, 1.59]	0.3	Not applicable		
	NMES	WB-NMES	2 (79)	−0.07 [−0.65, 0.52]	0.83	0	<0.01	93.3
		WB-NMES&ns	3 (182)	−2.98 [−4.34, −1.62]	<0.01	85		
weight	exercise	RT	3 (231)	−0.44 [−1.57,0.69]	0.45	0	0.03	78.7
		MT	5 (220)	−4.79 [−8.55, −1.02]	0.01	84		
	nutrition	HP	4 (212)	0.17 [−2.74, 3.09]	0.91	0	0.77	0
		ONS	2 (145)	−0.34 [−2.38, 1.69]	0.74	0		
BMI	exercise	RT	3 (231)	−0.18 [−0.68, 0.31]	0.47	0	0.04	77.3
		MT	4 (140)	−1.1 [−1.99, −0.39]	<0.01	0		
	nutrition	HP	4 (351)	−0.07 [−0.55, 0.41]	0.78	27	0.79	0
		ONS	1 (115)	0.42 [−3.23, 4.07]	0.82	Not applicable		
FM	exercise	RT	2 (81)	−1.25 [−1.40, −1.10]	<0.01	0	<0.01	91.7
		MT	3 (170)	−10.39 [−15.56, −5.22]	<0.01	95		
	nutrition	HP	5 (316)	0.16 [−1.07, 1.39]	0.8	0	0.16	48.3
		ONS	2 (145)	−0.89 [−1.73, −0.05]	0.04	50		
TFFM	exercise	RT	2 (179)	0.74 [0.62, 0.87]	<0.01	0	0.51	0
		MT	5 (220)	2.27 [−2.27, 6.82]	0.33	96		
	nutrition	HP	4 (256)	0.68 [−0.24, 1.59]	0.15	0	0.74	0
		ONS	2 (145)	0.94 [−0.28, 2.16]	0.13	0		
SMI	exercise	RT	3 (132)	0.83 [0.20, 1.46]	0.01	13	0.06	71.6
		MT	2 (87)	0.14 [−0.22, 0.49]	0.45	0		
	NMES	WB-NMES	1 (50)	0.21 [0.12, 0.30]	<0.01	Not applicable	0.3	8.1
		WB-NMES&ns	2 (115)	0.26 [0.23, 0.30]	<0.01	53		
WC	nutrition	HP	3 (182)	1.01 [−1.07, 3.08]	0.34	54	0.45	0
		ONS	2 (145)	−0.67 [−4.49, 3.14]	0.73	77		
	NMES	WB-NMES	2 (79)	−1.41 [−2.60, −0.21]	0.02	0	0.54	0
		WB-NMES&ns	1 (67)	−1.84 [−2.58, −1.10]	<0.01	Not applicable		
ASM	nutrition	HP	2 (134)	0.79 [−0.56, 2.15]	0.25	24	0.84	0
		ONS	1 (115)	0.56 [−1.22, 2.34]	0.54	Not applicable		
BMD	exercise	RT	3 (139)	0.02 [−0.02, 0.07]	0.34	0	0.1	64
		MT	2 (102)	−0.02 [−0.03, −0.01]	<0.01	0		
Physical function indicators								
HS	exercise	RT	1 (46)	2.19 [1.81, 2.57]	<0.01	Not applicable	0.04	75.3
		MT	6 (249)	3.19 [2.29, 4.09]	<0.01	41		
	nutrition	HP	6 (409)	1.35 [0.43, 2.26]	<0.01	0	0.98	0
		ONS	2 (145)	1.36 [0.45, 2.28]	<0.01	0		
GS	exercise	RT	4 (180)	0.15 [−0.00, 0.31]	0.05	92	0.26	20.2
		MT	2 (120)	0.29 [0.10, 0.48]	<0.01	85		
1 RM	exercise	RT	1 (41)	33.10 [22.02, 44.18]	<0.01	Not applicable	0.68	0
		MT	2 (97)	21.40 [−32.20, 75.00]	0.43	86		
Physiological indicators								
TG	exercise	RT	1 (35)	−0.50 [−51.99, 50.99]	0.98	Not applicable	0.96	0
		MT	2 (78)	0.86 [−5.13, 6.86]	0.78	30		
	nutrition	HP	2 (134)	1.30 [−16.51, 19.10]	0.89	0	0.93	0
		ONS	1 (30)	−0.40 [−33.43, 32.63]	0.98	Not applicable		
	NMES	WB-NMES	2 (79)	−1.63 [−18.77, 15.52]	0.85	40	0.62	0
		WB-NMES&ns	1 (67)	−8.30 [−28.55, 11.95]	0.42	Not applicable		
TC	nutrition	HP	2 (134)	4.45 [−8.96, 17.86]	0.52	0	0.79	0
		ONS	1 (30)	0.00 [−30.48, 30.48]	1	Not applicable		
GLU	nutrition	HP	2 (134)	4.66 [0.43, 8.88]	0.03	0	<0.01	90.9
		ONS	1 (30)	−2.60 [−3.30, −1.90]	<0.01	Not applicable		
Hb	nutrition	HP	3 (227)	−0.39 [−0.78, −0.00]	0.05	0	0.69	0
		ONS	1 (30)	−0.20 [−1.06, 0.66]	0.65	Not applicable		
HOMA-IR	nutrition	HP	2 (124)	−2.02 [−7.41, 3.37]	0.46	98	0.47	0
		ONS	1 (40)	0.00 [−0.73, 0.73]	1	Not applicable		

Indicators: BF%: body fat percentage; BMI: Body Mass Index; FM: fat mass; TFFM: total fat-free mass; SMI: Skeletal Muscle Index; WC: waist circumference; ASM: appendicular skeletal muscle mass; BMD: bone mineral density; HS: handgrip strength; GS: gait speed; 1 RM: one-repetition maximum; TG: Triglycerides; TC: total cholesterol; GLU: glucose; Hb: hemoglobin; HOMA-IA: homeostatic model assessment of insulin resistance. Subgroup: WB-NMES: whole-body neuromuscular electrical stimulation; WB-NMES&ns: whole-body neuromuscular electrical stimulation and nutrition; RT: resistance training; MT: mixed training; HP: high protein; ONS: other nutritional interventions (vitamin D, probiotics).

## Data Availability

The data supporting the findings of this meta-analysis are derived from previously published studies, which are cited in the reference list.
